# Patient-Derived Tumor Chemosensitization of GKB202, an *Antrodia Cinnamomea* Mycelium-Derived Bioactive Compound

**DOI:** 10.3390/molecules26196018

**Published:** 2021-10-04

**Authors:** Tsung-Ju Li, Ting-Wei Lin, Shih-Pei Wu, Hsin-Tung Chu, Yu-Hsuan Kuo, Jeng-Fong Chiou, Long-Sheng Lu, Chin-Chu Chen

**Affiliations:** 1 Biotech Research Institute, Grape King Bio, Taoyuan 32542, Taiwan; tsungju.li@grapeking.com.tw (T.-J.L.); tingwei.lin@grapeking.com.tw (T.-W.L.); carol.chu@grapeking.com.tw (H.-T.C.); yuhsuan.kuo@grapeking.com.tw (Y.-H.K.); 2 CancerFree Biotech Ltd., Taipei City 106, Taiwan; shihpei.wu@cancerfree.io; 3 Department of Radiation Oncology, Taipei Medical University Hospital, Taipei City 110, Taiwan; solomanc@tmu.edu.tw; 4 TMU Research Center of Cancer Translational Medicine, Taipei Medical University, Taipei City 110, Taiwan; 5 Department of Radiology, School of Medicine, College of Medicine, Taipei Medical University, Taipei City 110, Taiwan; 6 Graduate Institute of Biomedical Materials and Tissue Engineering, College of Biomedical Engineering, Taipei Medical University, Taipei City 110, Taiwan; 7 International Ph.D. Program in Biomedical Engineering, College of Biomedical Engineering, Taipei Medical University, Taipei City 110, Taiwan; 8 International Ph.D. Program for Cell Therapy and Regenerative Medicine, College of Medicine, Taipei Medical University, Taipei City 11031, Taiwan; 9 Department of Food Science, Nutrition, and Nutraceutical Biotechnology, Shih Chien University, Taipei City 104, Taiwan; 10 Institute of Food Science and Technology, National Taiwan University, Taipei City 10617, Taiwan

**Keywords:** *Antrodia Cinnamomea* mycelium, ACM, circulating tumor cells, patient-derived organoid, 5-FU

## Abstract

Oral cancers, hepatocellular carcinoma, and colorectal cancers are the three most common cancers, leading to 18,000 cases of cancer-related mortality in Taiwan per year. To bridge the gap towards clinical translation, we developed a circulating tumor cell (CTC) organoid culture workflow that efficiently expands CTC from patients to test *Antrodia Cinnamomea* mycelium-derived bioactive compounds. Three ACM-derived bioactive compounds were evaluated for tumor chemosensitization characteristics. Significant and consistent cytotoxic/5-FU sensitizing effects of GKB202 were found on 8 different patient-derived tumors. Acute toxicity profile and hepatic metabolism of GKB202 in rats suggest GKB202 is rapidly cleared by liver and is well tolerated up to the dose of 20 mg/kg. This comprehensive study provides new evidence that liquid fermentation of *Antrodia cinnamomea* mycelium (*ACM*) contains bioactive compounds that lead to effective control of CTC, especially when combined with 5-FU. Together, these data suggest ACM-derived GKB202 may be considered for further clinical investigation in the context of 5-FU-based combination therapy.

## 1. Introduction

*Antrodia cinnamomea* (*AC*), also known as *Antrodia camphorata*, is a native traditionally used medicine in Taiwan [1]. It is a unique parasitic fungal species that grows slowly in the tree *Cinnamomum kanehirai* [2]. Previous studies have found *AC* mycelium (ACM) provides many beneficial pharmacological activities including anti-inflammation [3], anti-obesity [4], anti-cancer [5], reduction of mild hypertension [6] and improvement of nonalcoholic steatohepatitis [7,8]. Ethanolic extract of *AC* contains several bioactive constituents which display promising anti-tumor effects in several types of cancers [9,10,11]. A purified derivative of maleic and succinic acid, YMGKI-1, was previously discovered in those ACM ethanolic extracts which have shown inhibition in the stemness of head and neck cancer initiating cells both in vitro and in vivo [12]. Despite the increasing evidence that *AC* has the capability to diminish the stemness of cancer initiating cells through modulating tumor miRNA expressions [13,14,15], there are still great challenges to translate in vivo findings into current human clinical trials endpoints [16].

To bridge the gap towards clinical translation, testing ACM effects on human derived cancer cells is a promising approach. Such an approach provides a direct cytotoxic measure for naturally occurring human cancers and avoids introducing artifacts that are inherent to currently available cell lines. It has been recently described that organoid culture of patient-derived tumor cells correlated to clinical outcome in metastatic gastrointestinal cancers [17]. While surgical tumor sampling is usually required to establish patient-derived xenografts, technological breakthrough now allows “liquid biopsy” with circulating tumor cells (CTC).

CTC are cancer cells shed into blood stream from an established tumor. Cancer initiating cells in CTC can seed distal target organs, give rise to new tumor lesions, and contribute to cancer progression. Clinically, CTC count is inversely correlated with survival outcome in many tumor types, including cancers of breast, lung, colon, pancreas, and prostate [18]. In the blood steam, CTC with high stemness gene expression may form clusters, have higher metastasis-initiation capability [19], and carry higher risk for cancer-related death [20]. Therefore, it is tempting to speculate that drug treatments targeting these cluster-forming CTC may provide a precise treatment to curb the spread of cancers. 

**Scheme 1:**
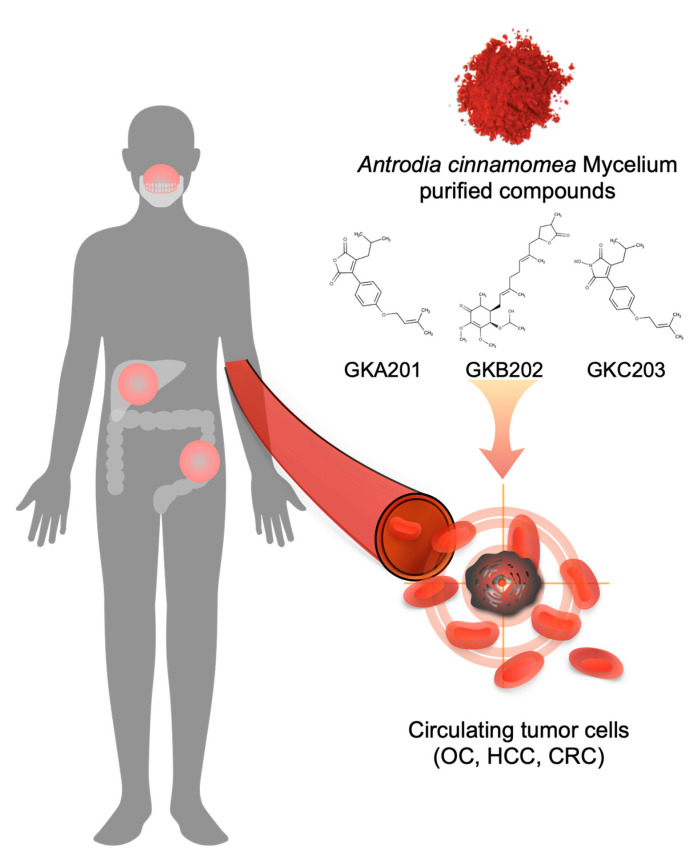
Three purified compounds from ACM extracts targeting circulating tumor cells. The circulating tumor cells were expanded from the oral cancer (OC), hepatocellular carcinoma (HCC) and colorectal cancer (CRC) patients.

Recently, our group developed a CTC organoid culture workflow that efficiently expand CTC from patients with extensive stage small cell lung cancer [21]. Moreover, the drug sensitivity profile from these CTC-derived organoids correlates well to the clinical treatment response. In this research (Scheme 1), we aim to characterize safety and metabolic stability of three ACM-derived bioactive compounds. Furthermore, we take advantage of CTC-derived organoids to test the drug sensitivity profile and chemosensitizing effects of these compounds in common gastrointestinal tract cancers including oral cancer, hepatocellular carcinoma, and colorectal cancer. These cancers are the three most common gastrointestinal tract cancers, leading to 18,000 cases of cancer related mortality in Taiwan per year. A novel approach to sensitize these cancers to existing therapeutics may have great health impacts both domestically and internationally.

## 2. Materials and Methods

### 2.1. Preparation of the Purified Compounds from A Specific Batch of ACM

*Antrodia cinnamomea* (*Antrodia camphorata*, BCRC 35398) strain was purchased from the Biological Resources Conservation and Research Center of the Food Industry Development Research Institute, Hsinchu, Taiwan. When *Antrodia cinnamomea* hyphae was grown on the PDA plate, 0.5 cm square bacterial clumps were taken, inoculated into a 2 L Erlenmeyer flask (containing 1 L liquid medium), and shaken at 25 °C and 100 rpm. They were cultivated for 2 weeks, then inoculated in a 500 L upgraded fermentation tank, and incubated for 7 days at 25 °C under stirring at 90 rpm and aeration of 0.5 vvm. They were then freeze-dried and ground to obtain the liquid fermentation mycelium powder of *Antrodia cinnamomea* (ACM). Liquid culture medium composition: 1.0% glucose, 0.5% soybean powder, 0.5% egg white, 0.3% (NH_4_)_2_SO_4_, 0.1% MgSO_4_∙7H_2_O. Freeze-dried ACM powder was analyzed using high-performance liquid chromatography (HPLC) and liquid chromatography-mass spectrometry (LC/MS) to evaluate the active peaks’ contents. HPLC analysis of GKA201, GKB202, and GKC203 in the ACM powder were performed using the following procedure: *Antrodia cinnamomea* mycelium powder was immersed in ethanol for 1 h and extracted three times. The extracts were concentrated under reduced pressure, the obtained extracts were sequentially mixed with n-hexane, dichloromethane, and n-butanol solvents. Partition separation extraction resulted in four layered extracts, which includes *n*-hexane layer, dichloromethane layer, *n*-butanol layer, and water layer. The dichloromethane layer extract was further applied to silica gels for column chromatography, using n-hexane and ethyl acetate as eluents. GKA201, GKC203, and GKB202 were obtained after eluting with 15% ethyl acetate, 20% ethyl acetate, and 50% ethyl acetate, respectively. After identifying precursor → product ion transitions, these compounds’ structures were confirmed using the liquid chromatography in combination with tandem MS (LC-QTOF/MS). For HPLC analysis, the retention time of GKA201, GKC203, and GKB202 were compared with the available standards according to previous methods [12,22].

### 2.2. Patients and CTC Expansion

Patients who were diagnosed with oral cancer (OC), hepatocellular carcinoma (HCC) and colorectal cancer (CRC) with stage III or IV were recruited and provided written informed consent to participate in this study. For patients enrolled, clinical profiles including initial cancer staging, medication information, and treatment responses were recorded. Peripheral blood (7.5 mL) was collected in vacated ethylenediaminetetraacetic acid (EDTA) tubes and peripheral blood mononuclear cells (PBMCs) were isolated by gradient gravity centrifugation using Ficoll-Paque. Blood was added to a LeucoSep tube filling with 15 mL of Ficoll-Paque Plus in the bottom of the frit and centrifuged to obtain the PBMC fraction. Then PBMC was resuspended in phosphate-buffered saline (PBS) containing 1% BSA, 2 mM EDTA where CTC was enriched by RosetteSep^®^ CTC Enrichment Cocktail kit. Briefly, the PBMC pellet is incubated with antibodies at room temperature, followed by the addition of PBS containing 2% FBS to the diluted sample. The diluted sample was added on the top of Ficoll-Paque for another centrifugation, then enriched cells were obtained and suspended in DMEM/F12 medium containing EGF, bFGF, and B27 supplement. Cells were seeded onto a substrate of binary colloidal crystals (BCCs). The structure of BCCs has been reported for its ability to enhance the expansion of rare cancer cells [23]. The medium was replaced in each well every four days. Tumor-derived CTC spheroids were checked by optical microscopy.

### 2.3. Viability Assay and the Screening of Anti-Tumor Activity of ACM-Derived Compounds

To test the anti-tumor activity of natural compounds, CTC spheroids were allowed to grow for four weeks then spheroids were collected to seed into other BCC plates and incubated for 16 h. Two chemotherapy drugs used as the positive control including 5-FU (30 µM), Cisplatin (14.4 µM), GKA201, GKB202 and GKC203 (4 µg/mL and 20 µg/mL) were added to cells and incubated for six days. Cell viability was determined by a CellTiter-Glo^®^ Luminescent Cell Viability Assay (Promega Corporation, Madison, WI, US) where cells were incubated with a Cell-Titer Glo reagent for 10 min and the luminescence was read on a GloMax^®^ Navigator Microplate Luminometer (Promega Corporation, Madison, WI, US). The relative cell viability was calculated as the percentage of cells treated with drugs or natural compounds compared with untreated control cells.

### 2.4. Viability Assay and the Screening of Anti-Tumor Activity of GKB202 Compound

All the animal experiments in this study were conducted by a third party preclinical CRO Super Laboratory Co., Ltd. (New Taipei, Taiwan) and the animal use protocol has been approved by the Institutional Animal Care and Use Committee (approval number 110-1i, Figure S2). The Sprague-Dawley rats used in this study were obtained from BioLASCO (Taiwan Co., Ltd., Taipei, Taiwan). In the *Antrodia cinnamomea* mycelium GKB202 compound experiments, three different doses (5, 10 and 20 mg/kg BW) were orally administered.

### 2.5. Bioactivation Analysis of GKB202 in Rat Liver S9

According to the manufacturer recommendations of the S9 and regeneration system (MOLTOX^®^, Lot no.: 4385, US), 1 mg/mL of rat liver S9 fraction is prepared with the NADPH Regensys system (MOLTOX^®^, Lot no.: 21080RA, US) and stored on ice before supplementing with the GKB202 compound. The GKB202 is added to the stock solution with a final concentration of 10 µM in 1 mL. The solution is then activated by incubation in a 37 °C water bath for metabolic reaction. The reaction time is 0, 10, 30, 60, and 180 min. When the reaction time is up, 150 μL of the solution is aliquoted and cold acetonitrile (ACN) was used to stop the reaction.

### 2.6. Quantification of GKB202 Metabolic Stability in Rat Liver S9

High performance liquid chromatography (HPLC model: 1100, Agilent, Palo Alto, CA, USA) was coupled with a triple quadrupole mass spectrometer (QQQ/MS model: API 3000, Applied Biosystem, Warrington, UK). Data acquisition and processing was performed by Analyst^®^ 1.5.1 software (Applied Biosystem, Ontario, Canada). The chromatographic separation was achieved on a 3.5 µm Agilent Eclipse XDB-C18: 100 mm × 4.6 mm i.d. (Waters, Milford, MA, USA) at 22 °C. Mobile phase consisted of water for phase A and methanol for phase B. Separation was optimized using a gradient method with mobile phase A/B set to 95%/5% from 0.00 to 5.00 min and 0%/100% from 5.00 to 10.00 min and then back to 95%/5% from 13.50 to 15.00 min.; flow rate was 300 μL/min; sample injection volume was 10 μL. The mass spectrometer was operated in the positive ion mode. The nebulizer gas, curtain gas, collision gas, ion spray voltage and source temperature were set at 8 psi, 7 psi, 2 psi, 4500 volts, and 350 °C, respectively. The precursor-product ion pairs used for multiple reaction monitoring (MRM) of GKB202 were *m/z* 485.2 → 425.3 (declustering potential, 86; focusing potential, 270; collision energy, 17; collision cell exit potential, 18), *m/z* 485.2 → 137.0 (declustering potential, 46; focusing potential, 160; collision energy, 29; collision cell exit potential, 4).

### 2.7. Statistical Analysis

All data collections with indicated sample sizes are indicated by the mean ± standard deviation (SD). Graphpad Prism 8.4 was used for graph output and statistical analysis. This study used one-way analysis of variance (ANOVA) to measure the difference between each group with a post-hoc comparison. * Signs indicating a statistically significant difference with *p* < 0.05.

## 3. Results

### 3.1. Fingerprint Chromatogram of Pure Compounds from the Powder of ACM

**Figure 1:**
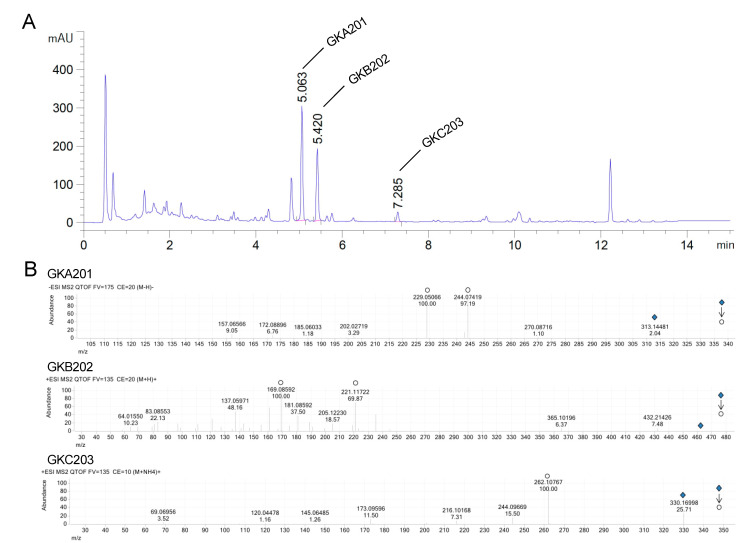
(**A**) HPLC of purified GKA201, GKB202, and GKC203 from ACM with retention time labeled at 5.063 for GKA201, 5.420 for GKB202, 7.285 min for GKC 203. (**B**) LC-QTOF/MS spectra of GKA201 with parent ion detected at *m/z* 313.14481; GKB202 with parent ion detected at *m/z* 463.26310; GKC203 with parent ion detected at *m/z* 330.16998.

The fingerprint chromatogram that contains three specific active components were purified from the liquid fermentation of *Antrodia cinnamomea* mycelium (Figure 1A). The chemical structures of GKA201, GKB202 and GKC203 were compared with their standard by their retention time at 5.063, 5.420 and 7.285 min, respectively. These compounds’ structures were confirmed using liquid chromatography in combination with tandem MS (LC-QTOF/MS) operating in multiple reaction monitoring (MRM) mode by identifying precursor → product ion transitions (Figure 1B).

### 3.2. Establishing Patient Derived Circulating Tumor Cells Profile

**Figure 2:**
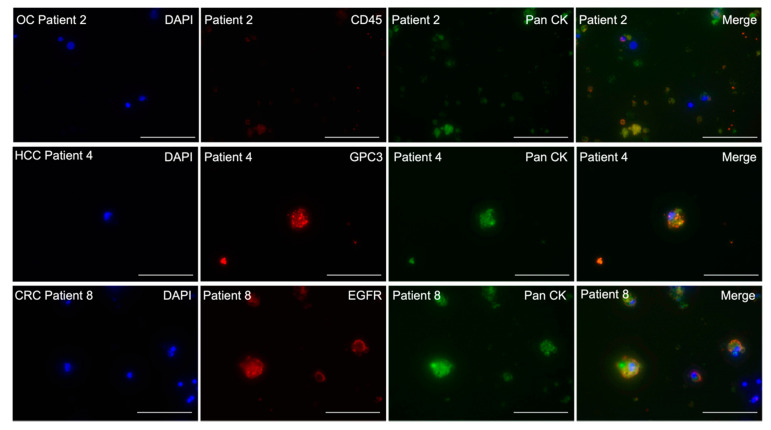
Immunofluorescence analysis of expanded circulating tumor cells. OC: oral cancer; HCC: hepatocellular carcinoma; CRC: colorectal cancer. PanCK is a cancer-specific surface marker. CD45, GPC3, EGFR are markers specific to leucocytes, HCC, and CRC, respectively. Representative images from one patient in each cancer type is shown. The scale bar is 75 μm in length.

A total of 8 cancer patients’ blood was collected and enriched according to the previously published method [21]. According to Table 1, patients with one each of the three stage III or IV cancers (OC: oral cancer, HCC: hepatocellular carcinoma, CRC: colorectal cancer) were selected. All patients’ CTCs cellular metabolic activities were calculated and quantified using the CellTiter-Glo^®^ Luminescent Assay. Immunofluorescent staining of isolated cells was stained by triple color staining DAPI+/CD45−/pan-cytokeratin (panCK)+ protocol [24] to identify the CTCs surface marker profile (Figure 2; Patient 2 as representative). Besides (OC) group, Glypican-3 (Gpc3) was additionally stained as a common overexpressed marker for hepatocellular carcinoma (HCC) patients (Figure 2; Patient 4 as representative). Whereas for colorectal cancer (CRC) patient-derived CTCs, epidermal growth factor receptors (EGFR) were additionally stained to observe their expression (Figure 2; Patient 8 as representative). The rest of the patient immunofluorescence data can be found in Supporting Information (Figure S1). Results show that all patient-derived CTCs were established and quantified before the compound treatments.

### 3.3. Effect of GKA201, GKB202 and GKC203 on Patient-Derived Circulating Tumor Cells Survival Rate

**Figure 3:**
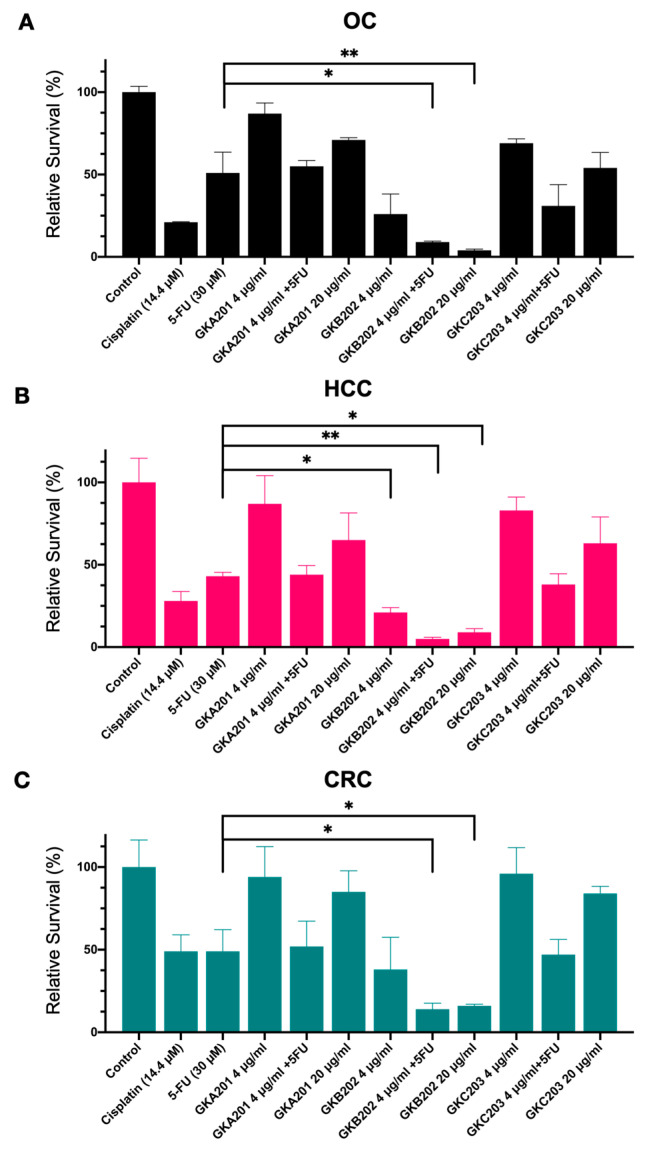
Ex vivo expansion toxicity assay with different purified AC compounds at low and high dosage (4 and 20 µg/mL respectively) in different cancer types. Each patient-derived CTC was tested three times for different assay groups. (**A**) OC: oral cancer, total patients, *N* = 2; (**B**) HCC: hepatocellular carcinoma, total patients, *N* = 3; (**C**) CRC: colorectal cancer, total patients *N* = 3. * Represents a significant difference *p* < 0.05 and ** represents a significant difference *p* < 0.01.

To evaluate the ACM purified compound for anti-CTC efficacy, viable patient-derived circulating tumor cells were treated at low dose (4 µg/mL) and high dose (20 µg/mL). Figure 3 shows the positive control drug treatments, Cisplatin at 14.4 µM has higher inhibition in both the oral cancer (21 ± 0.3%) and hepatocellular carcinoma groups (28 ± 5.8%). However, the treatment is less effective for colorectal cancer (49 ± 10%). In contrast, 5-FU presented a substantial survival rate in all cancer types at 30 µM. In brief, the relative survival rate for OC is 51 ± 13%, HCC is 43 ± 2% and CRC is 49 ± 13%. As for compound comparisons in different cancer groups, GKA201 and GKC203 showed a dosage dependent trend but indicated no significance when compared with the 5-FU group. In contrast, GKB202 treatment showed significant anti-CTC properties in all cancer types at high dose 20 µg/mL when compared with the 5-FU group. Furthermore, the synergistic effect of anti-CTCs can be found when combining low dosage of GKB202 with 5-FU. In brief, this excellent anti-CTC effect has significantly lowered the relative survival rate in OC at 9 ± 1%, HCC is 5 ± 1% and CRC is 14 ± 4% (*p* < 0.05).

### 3.4. In Vivo Observation of GKB202 in Sprague-Dawley Rat Analysis

Based on the most potent anti-CTC drug candidate in this study, purified compound GKB202 was further analyzed using acute oral toxicity tests for future preclinical safety. Results show all experimental rats survived up to 20 mg/kg before autopsy analysis (Table 2). At the highest dosage, the organ remains normal with no abnormality observed. The body weight showed no adverse effect in all rats with a consistent body weight gain. Overall, all GKB202 treatment groups did not show significant weight changes when compared with the control (Table 2). The biochemical analysis of the blood samples is also shown in Table 2. In addition, AST and ALT levels showed no notable changes up to 20 mg/kg after 1 week of recovery when compared with the control group.

### 3.5. Metabolic Stability of GKB202 in Rat Liver S9 Analysis

**Figure 4:**
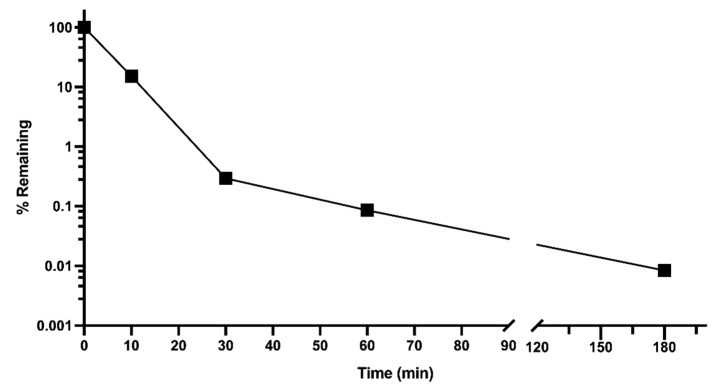
GKB202 remaining versus time profile in SD rat Liver S9. Data is presented in mean values of triplicate measurements.

To further understand the GKB202 metabolic stability, GKB202 was examined using liver S9 fractions of the animal. The S9 fraction system is a widely used in vitro analysis to study drug metabolism. In the presence of the NADPH, GKB202 was metabolized rapidly within 30 min (0.2%) with T_1/2_ = 3.68 min and down to less than 1 ppb after 60 min (Figure 4). Using the ‘well-stirred model’, the predicted value showed that GKB202 has a high intrinsic hepatic clearance value with an extraction value (E_H_) value at 0.95 (Table 3).

## 4. Discussion

AC extracts have long been explored for their potential anticancer effects. The safety profile of AC extracts is generally well tolerated in patients with advanced cancers. In this paper, we purified three bioactive compounds, GKA201, GKB202 and GKC203, from liquid fermentation of AC mycelium. Consistent and significant cytotoxic/5-FU sensitizing effects of GKB202 were found on 8 circulating tumor cell organoids from patients with oral cancers, hepatocellular carcinoma, and colorectal cancers. Further studies on the acute toxicity profile and hepatic metabolism of GKB202 in SD rats suggest GKB202 is rapidly cleared by the liver and is well tolerated up to a dose of 20 mg/kg. Collectively this is a safe and novel ACM-derived bioactive compound with a potential towards further clinical development.

In parallel with the present study, we have tested MTT and annexin V/PI stains when establishing the protocol. However, these technologies did not perform as accurately and reproducibly for the small amount of CTC typically present in a test aliquot (~1000–3000 cells). Therefore, we concluded that technically it was not feasible to measure the viability via annexin V/PI staining when analyzing patient-derived CTC for drug sensitivity profiling. In contrast, in the current spheroid culture system, patient-derived organoids (PDO) retain genomic, transcriptomic, and biological characteristics as well as tumor heterogeneity of the original tumors better than established cell lines [25,26]. Moreover, the availability of clinical data for these cells allows precise assessments of unique clinical scenarios, such as resistance to commonly used chemotherapeutics. This makes PDO a promising drug screening platform [26]. Most patient-derived CTCs in this study (Figure 2) have expressed a typical surface marker PanCK+/CD45− which is like other reports [27]. The CTC organoids used in this research were found with intrinsic resistance to 5-FU (43–51% of survival comparing with untreated controls). While clinical CTC studies have shown that the drug sensitivity is highly concordant with the patients’ treatment response [28], our findings are consistent with the fact that these organoids were derived from patients with treatment-resistant stage III or IV diseases (Figure 3). It is worthwhile to mentioned that recruited OC and CRC patients have failed 5-FU treatment as a standard first line. In contrast, HCC patients were not exposed to 5-FU. This result demonstrates that current finding of GKB202 can show effective inhibition of even those tumor cells that may have undergone genomic changes. In addition, the current platform has also shown that OC patient organoids are sensitive to Cisplatin but resistant to Carboplatin at the same concentration, showing the importance of personalized medicine (Figure S3).

AC extracts have long been explored for their potential anticancer effects and suppression of the multi-drug resistance gene [14]. Among terpenoids, benzoquinones, and polysaccharides that have been identified from extracts of AC in different stages, induction of mitochondrial apoptotic machinery [29], enhanced NF-kappaB activities [30], HDAC1-dependent histone hypoacetylation [31], and poly(ADP-ribose) polymerase proteolytic cleavage [32] are involved in their cytotoxic mechanisms at the range between 10–300 μg/mL. Interestingly, ACM extract also appears to be involved in selective inhibition of cancer stem cells via STAT3 inhibition and down-regulated Src signaling, which suppresses epithelial growth factor-induced cancer stem cell conversion and inhibits tumorigenicity in vivo [33]. The broad impacts of ACM extracts in cancer signaling and invasiveness make it a rich repertoire of potential botanical therapeutics.

In the clinical setting, there are studies to explore the potential efficacy of AC extracts in cancer patients. Durable tumor control has been reported with AC extracts in patients with small cell lung cancer [34]. In a clinical trial enrolling patients with advanced cancers, the use of AC extract also has a trend towards disease control and clinical efficacy [35]. This is consistent with what we have found in the current study with 8 CTC organoids from patients with oral cancer, hepatocellular carcinoma, and colorectal cancer. These organoids showed lineage specific markers in concordance with their tumor origin (pancytokeratins and glypican 3) and were depleted of myeloid and lymphoid cells as shown by the lack of CD45 stains. Comparing to cisplatin and 5-FU, the ACM-derived compounds GKB202 showed at least comparable cytotoxic profiles. GKB202 is especially impressive in its cross-spectrum cytotoxicity in both CRC and HCC cancer types. Interestingly, we noticed that GKB202 also significantly synergizes with 5-FU. Chemosensitization from AC extracts is a commonly proposed mechanism of action in clinical observations [34]. The drug 5-FU, a commonly used chemotherapeutic for gastrointestinal cancers and in head and neck cancers, inhibits thymidylate synthase and interrupts pyrimidine thymidylate biosynthesis, which is an essential substrate for DNA replication. The resultant lack of thymidine triphosphate leads to a stop in the DNA replication process and subsequent death of rapidly proliferating cancer cells. Targeting alternative survival pathways via STAT3/src signaling and activating mitochondrial apoptosis have been proposed to synergize with genotoxic action of 5-FU for chemosensitization [36,37]. This synergetic effect combined with 5-FU can also be found in other natural compounds derived from honey for colorectal cancer treatment with anti-metastatic effects [38,39]. Overall, this is consistent with the potential mechanism of action for ACM-derived compounds as observed in our studies.

To further understand the safety of GKB202 compounds, no significant adverse effects were found as high as 20 mg/kg in oral supplements (Table 2). No statistical differences were observed although there was a slight body weight loss. While liver is the vital organ for drug metabolism, we also include the biochemistry analysis of AST and ALT (Table 2) and compared it with the control. All value falls under normal range when compared with other SD rat toxicity studies [40]. Finally, using the in vitro rat S9 liver analysis (Figure 4), the GKB202 was predicted to have a high CL_H_ value close to hepatic blood flow (Table 3). This may explain the safety of high dose supplements due to the high hepatic ratio. A recent case report has shown that a combination of existing therapeutic drugs with AC (10 g/day) in a breast cancer patient with bone metastasis showed life quality improvement by decreasing CTC numbers [41], with no further adverse effect found in both liver and renal after a long period of consumption. Since GKB202 is a purified compound from ACM, its metabolites should be analyzed and compared with ACM metabolites to monitor potential biomarkers in future work. By understanding GKB202 metabolites in vivo pharmacokinetic study, nephrotoxic study, and patient CTC organoids at different stages, it will be possible to achieve an optimal pharmacological profile for future clinical translation.

## 5. Conclusions

In summary, this comprehensive study provides new evidence that *Antrodia cinnamomea* mycelium (ACM) content contains bioactive compounds that are not only beneficial for anti-cancer and anti-cancer stem cells but also favorable for anti-circulating tumor cells. We not only provided the metabolic profile of the potential compound GKB202, but for the first time provided evidence of synergy between 5-FU and GKB202 in patient-derived CTC organoids. Together, these data suggest ACM-derived compounds can be considered for further clinical investigation in the context of 5-FU based combination therapy for late-stage cancers.

## Figures and Tables

**Table 1:** Characteristics of cancer patient-derived CTCs.

Patient No.	Cancer Types	Pathological Stages	Gender	Age
**1**	OC	III	Male	40
**2**	OC	IVa	Male	60
**3**	HCC	III	Male	54
**4**	HCC	III	Male	66
**5**	HCC	IV	Male	62
**6**	CRC	IV	Female	47
**7**	CRC	IV	Female	66
**8**	CRC	IV	Male	56

**Table 2:** In vivo observation after GKB202 compound supplementation in different dosage.

GKB202	In Vivo Observation			
	Control	5 mg/kg	10 mg/kg	20 mg/kg
**SD Rat Status**				
Survival	5/5	5/5	5/5	5/5
Organ Abnormality	none	none	none	none
**Body Weight**				
Day 0 (g)	196.5 ± 10.6	203.4 ± 7.1	207.2 ± 3.5	205.0 ± 2.6
Day 8 (g)	214.6 ± 13.0	221.2 ± 12.5	221.3 ± 7.7	217.2 ± 3.0
**Biochemistry**				
AST (U/L)	74.8 ± 5.9	73.8 ± 7.2	76.2 ± 4.0	76.4 ± 2.6
ALT (U/L)	43.4 ± 7.3	41.6 ± 10.7	38.2 ± 5.7	41.4 ± 11.8

Data presented as mean ± SD with *N* = 5.

**Table 3:** Hepatic metabolic stability of GKB202 prediction in SD rat with presence of NADPH.

Species	T_1/2_ (min)	CL_int_ (mL/min/kg)	CL_H_ (mL/min/kg)	E_H_
**Rat**	3.68	1023.26	52.19	0.95

## Data Availability

The raw materials and compounds presented in this study are available from the corresponding author upon request.

## Supplementary Materials

The following are available online, Figure S1: Immunofluorescence of patient isolated circulating tumor cells with specific surface markers; Figure S2: Institutional Animal Care and Use Committee Approval for this study using purified compound from ACM; Figure S3: Ex vivo expansion toxicity assay with different chemical compounds for OC patient organoids. Each patient-derived CTCs were tested three times for different assay group.

## Sample Availability

Samples of the compounds are available from the authors.
