# Supplementary material for: Patient-Derived Tumor Chemosensitization of GKB202, an Antrodia Cinnamomea Mycelium-Derived Bioactive Compound
Source: Molecules. 2021 Oct 4;26(19):6018. doi: 10.3390/molecules26196018 (PMC8512555; doi:10.3390/molecules26196018)
Supplement: Supplementary file 1 [file molecules-26-06018-s001.zip › molecules-1365819-supplementary.pdf]

Supporting Information

Safety, Stability and Patient-Derived Tumor Chemosensitization of GKB202, An *Antrodia Cinnamomea* Mycelium-Derived Bioactive Compound

Tsung-Ju Li[1], Ting-Wei Lin[1], Shih-Pei Wu[2], Hsin-Tung Chu[1], Yu-Hsuan kuo[1], Jeng-Fong Chiou[3,4,5], Long-Sheng Lu[3,5,6,7,8,*], Chin-Chu Chen[1,9,10,*]

1 Biotech Research Institute, Grape King Bio, Taoyuan 32542, Taiwan

2 CancerFree Biotech Ltd., Taipei City 106, Taiwan

3 Department of Radiation Oncology, Taipei Medical University Hospital, Taipei city 110, Taiwan

4 TMU Research Center of Cancer Translational Medicine, Taipei Medical University, Taipei city 110, Taiwan

5 Department of Radiology, School of Medicine, College of Medicine, Taipei Medical University, Taipei, Taiwan

6 Graduate Institute of Biomedical Materials and Tissue Engineering, College of Biomedical Engineering, Taipei Medical University, Taipei city 110, Taiwan

7 International Ph. D. Program in Biomedical Engineering, College of Biomedical Engineering, Taipei Medical University, Taipei city 110, Taiwan

8 International Ph.D. Program for Cell Therapy and Regenerative Medicine, College of Medicine, Taipei Medical University, Taipei 11031, Taiwan.

9 Department of Food Science, Nutrition, and Nutraceutical Biotechnology, Shih Chien University, Taipei, Taiwan

10 Institute of Food Science and Technology, National Taiwan University, Taiwan

* Correspondence: lslu@tmu.edu.tw (L.-S.L);drccchen@g2.usc.edu.tw (C.-C.C.)

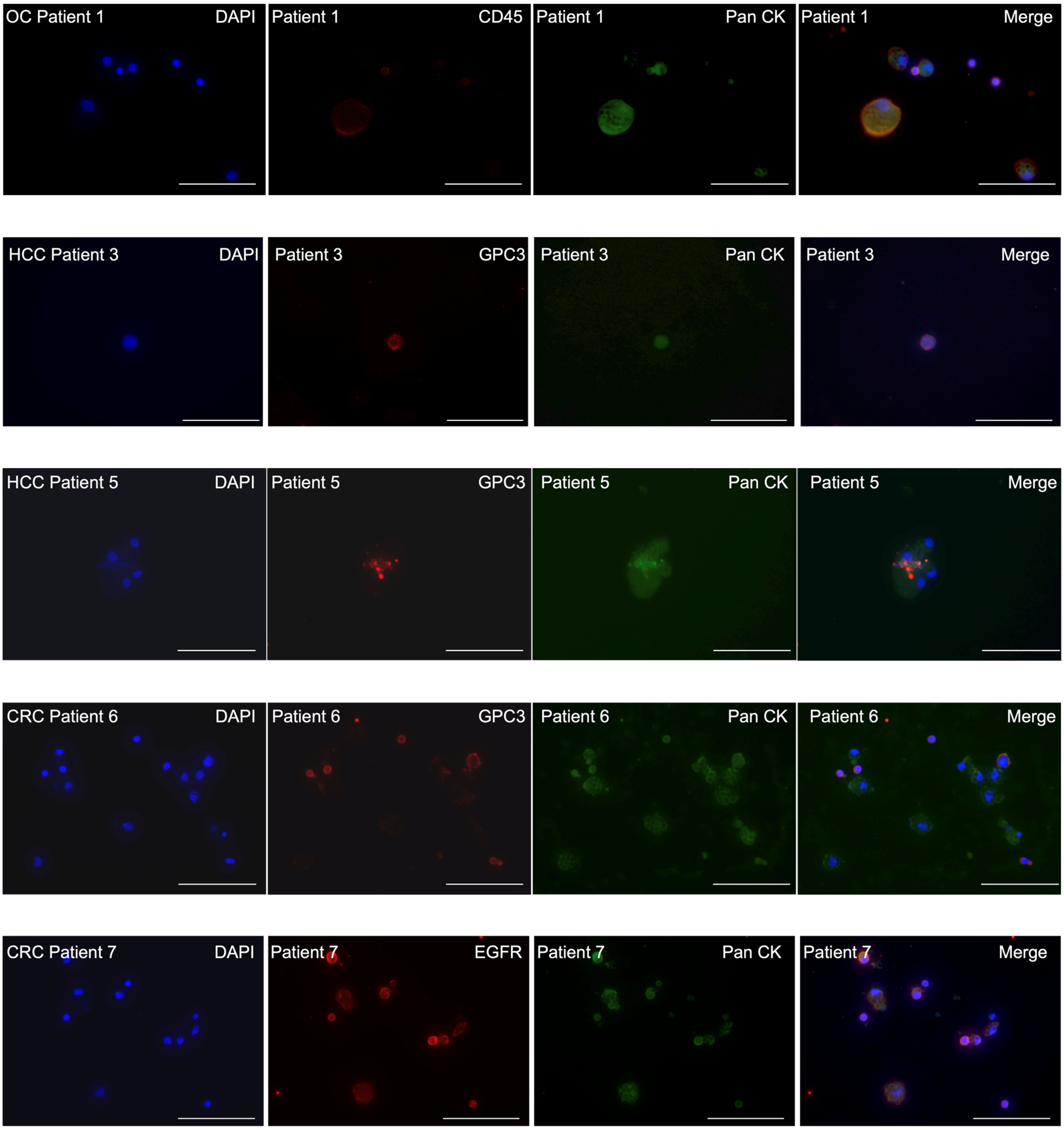

Figure S1. Immunofluorescence of patient isolated circulating tumor cells with specific surface markers. Patient no.1 represent oral cancer, patient no. 3 and no.5 represent hepatocellular carcinoma, patient no.6 and no.7 represent colorectal cancer. The scale bar is 75 μm in length.

台美檢驗科技股份有限公司
實驗動物照護及使用委員會審查同意書
Affidavit of Approval of Animal Use Protocol
SUPER LABORATORY

動物實驗核准編號（IACUC No.）： 110-1i

計畫申請人： 林嘉儀　　職 稱： 分析師
Principle Investigator（PI） Jia-Yi Lin　　Professional Title Analyst
單 位： 台美檢驗科技股份有限公司 委託研究實驗室
Corporation Super Laboratory Co., Ltd. Contract Research Organization
飼養/應用地點： 台美實驗動物中心
Address of Animal Facility Laboratory Animal Center of Super Laboratory Co., Ltd.

計畫名稱： 口服急性毒性試驗 –AC-1、AC-2、AC-3、AC-4、AC-5
Protocol Title Acute Oral Toxicity Test –AC-1、AC-2、AC-3、AC-4、AC-5

本計畫之「動物實驗申請表」經實驗動物照護及使用委員會■ 實質 □ 形式 審查通過
The animal experiment of this protocol approved by the Institutional Animal Care and Use Committee through ■substantive □formal review.
本計畫預定飼養應用之動物如下：
Expected feed animals of the protocol are shown in the following

動物種類 Animal Species	動物數量 Animal Amount	計畫執行期間 yyyy/mm/dd
SD 品系大鼠 Sprague Dawley Rat	雌 30 隻 (30 females)	2021/04/08～2021/05/10

*共購買 30 隻動物，30 隻用於正式試驗。
試驗結束時，試驗動物犧牲。動物屍體依台美標準作業程序 SOPP-321 進行處理。
* A total of 30 animals will be purchased, 30 animals will be used for main study. Rats will be sacrificed at the end of the test. Carcasses will be processed according to SuperLab standard operating procedure (SOPP-321).

The animal use protocol listed below has been reviewed and approved by the Institutional Animal Care and Use Committee（IACUC）.

實驗動物照護及使用委員會召集人　　日期
IACUC Chairman 林[illegible] Chia-hung Lin　Date 2021/03/05

Figure S2. Institutional Animal Care and Use Committee Approval for this study using purified compound from ACM.

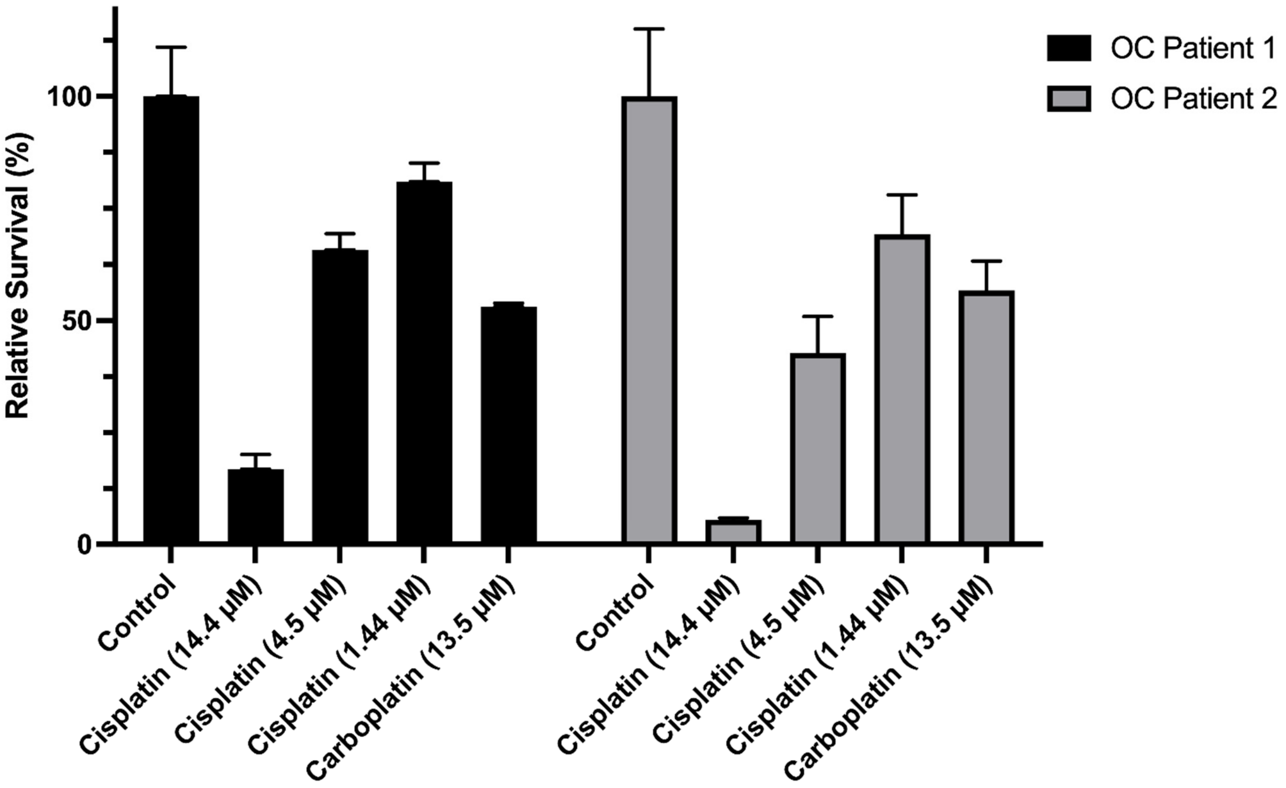

Figure S3. *Ex vivo* expansion toxicity assay with different chemical compounds for OC patient organoids. Each patient-derived CTCs were tested three times for different assay group.
